# Supplementary material for: Cryptic diversity and multiple origins of the widespread mayfly species group Baetis rhodani (Ephemeroptera: Baetidae) on northwestern Mediterranean islands
Source: Ecol Evol. 2016 Oct 11;6(21):7901–10. doi: 10.1002/ece3.2465 (PMC6093170; doi:10.1002/ece3.2465)

**Supporting information**

**Table S1.** PCR primers and annealing temperatures used to amplify the two mitochondrial and the two nuclear

DNA fragments used in this study.

| **Gene** | **Primer name** | **Primer sequence 5'-3'** | **Annealing T. (°C)** | **Source** |
| --- | --- | --- | --- | --- |
| CO1 | C1-N-2191 | CCCGGTAAAATTAAAATATAAACT TC | 56 | Simon et al. 1994 |
|  | C1-J-1718 | GGAGGATTTGGAAATTGATTGTTCC |  |  |
| ND1 | ND1F | TAAAGTTAGCAGGTTCATACCC | 58 | This study |
|  | ND1R | CACCTATATTTGTACTTTGAAGG |  |  |
| PEPCK | PepFb12 | GGAACTTCAAACAGCACCAAT | 53 | Pereira-da-Conceicoa et al. 2012 |
|  | PepRb45 | ACCTTGTGTTCTGCAGCT |  |  |
| RYA | RYAf | GGCGAGAAGAACATGCACG | 60 | This study |
|  | RYAr | CCAGTAGAAGTCCATGATGG |  |  |

**Figure S1.** Bayesian phylogenetic tree, estimated using MRBAYES, based on the CO1 haplotypes found in this study, and 2 representatives of each main clade previously identified by Williams et al., (2006) Lucentini et al., (2011), and Murria et al., (2014). Posterior probabilities are shown at the nodes when ≥0.90.

**
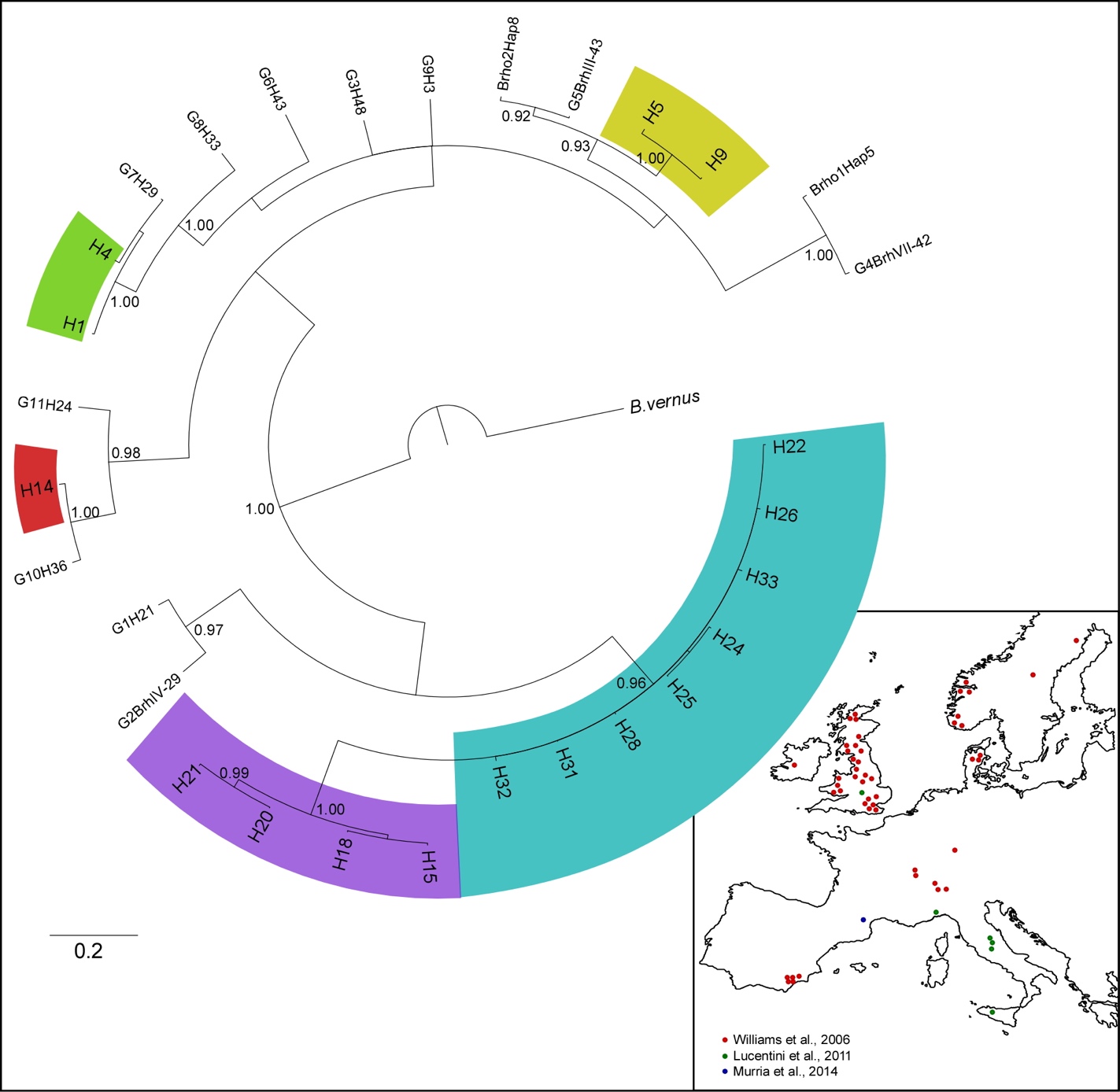
**

**Figure S2.** Maximum clade credibility multilocus species-trees obtained with *BEAST. Horizontal grey bars are 95% highest posterior densities of the estimated node ages. X-axis is in units of million years. All posterior probabilities were ≥0.99. (A) Analysis run with 5 terminal taxa, following results based on the concatenated mtDNA dataset (see main text Figure 1) (B) Analysis run with 4 terminal taxa, following results with BAPS based on nDNA data.


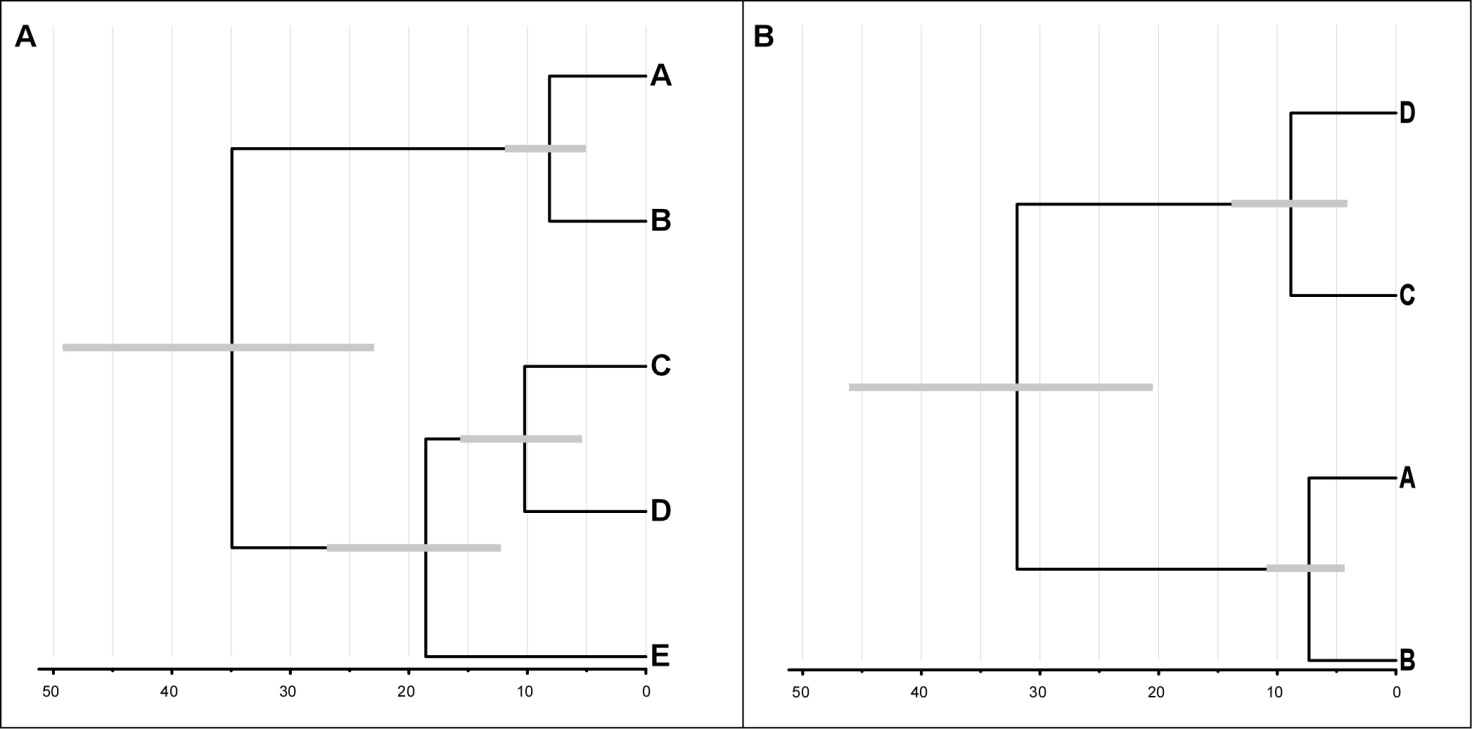

Supplement: Supplementary file 1 [file ECE3-6-7901-s001.docx]
